# Lignin-Based Adhesives for Various Packaging Applications

**DOI:** 10.3390/polym18151905

**Published:** 2026-08-03

**Authors:** Urška Klenovšek, Urška Vrabič-Brodnjak

**Affiliations:** University of Ljubljana, Faculty of Natural Sciences and Engineering, Department of Textiles, Graphic Arts and Design, Aškerčeva 12, SI-1000 Ljubljana, Slovenia; urska.klenovsek@ntf.uni-lj.si

**Keywords:** polyphenolic biopolymer, bio-based adhesives, packaging, packaging adhesives, sustainability

## Abstract

Growing demand for more sustainable packaging has increased interest in bio-based adhesives as alternatives to conventional fossil-derived systems. Among renewable raw materials, lignin is particularly attractive because of its aromatic structure, phenolic functionality, and availability as a side stream of pulp, paper, and biorefinery processes. This review critically examines the potential of lignin-based adhesives for packaging applications. Selected polysaccharide-, protein-, vegetable-oil-, and tannin-based systems are briefly discussed as comparative references, while the main focus is placed on the properties of kraft lignin, lignosulfonates, organosolv lignin, and soda lignin. Their structural differences, adhesive behaviour, and suitability for chemical modification through hydroxymethylation, phenolation, demethylation, depolymerization, oxidation, and epoxidation are evaluated. Because most lignin-adhesive research concerns wood bonding, the transferability of these findings to paper, paperboard, labels, coatings, films, and hot-melt packaging adhesives is critically assessed. Direct evidence for packaging applications remains limited, although existing studies demonstrate promising opportunities for paper bonding, pressure-sensitive systems, and paperboard hot-melt adhesives. Key challenges include lignin heterogeneity, processing complexity, moisture resistance, colour, food-contact safety, scalability, and compatibility with recycling.

## 1. Introduction

Packaging plays a crucial role in modern society by protecting and preserving products during transportation and storage against physical, chemical, and environmental stresses. Beyond these functional requirements, increasing attention is being directed toward the sustainability of packaging systems. This shift concerns not only primary packaging materials but also auxiliary components such as adhesives, which are essential for maintaining structural integrity and overall package performance [1].

Adhesives function as bonding agents that join substrates through interfacial interactions and cohesive strength within the adhesive layer. They are used across a wide range of industries, including construction, transportation, electronics, wood-based materials, packaging, and emerging biomedical technologies [2]. The global bio-based adhesive market is projected to grow at a compound annual growth rate (CAGR) of 3.5% from 2024 to 2030, reaching an estimated value of USD 2.8 billion [3]. This rapid growth highlights the critical role of adhesives in modern industrial systems, including the packaging sector.

At present, many commercial adhesives are derived from petrochemical resources, including urea–formaldehyde, phenol–formaldehyde, and melamine–formaldehyde systems. These materials offer advantages such as high-water resistance, strong adhesion performance, and well-established industrial processing. However, they also present significant drawbacks, particularly emissions of volatile organic compounds (VOCs) and formaldehyde, which pose risks to environmental and human health. In addition, their fossil-based origin and limited compatibility with recycling processes hinder the development of fully sustainable and circular packaging solutions [2].

As petrochemical resources continue to deplete and environmental awareness increases, there is growing interest in the development of bio-based adhesives derived from renewable biomass. Current research efforts are largely focused on partially or fully replacing formaldehyde-based systems with bio-derived alternatives. Among the most promising biomass resources are polysaccharides (e.g., starch and cellulose), proteins, tannins, and lignin [4].

Among renewable adhesive feedstocks, polysaccharides and proteins have received considerable attention because of their availability, film-forming ability, biodegradability, and compatibility with paper-based substrates [3,4]. However, many unmodified systems remain sensitive to moisture and may exhibit limited storage stability, slow drying, or insufficient durability under demanding service conditions. Chemical modification, crosslinking, and blending can improve their performance, but may also increase formulation complexity and reduce some of the advantages associated with their natural origin [5,6]. These systems nevertheless provide useful benchmarks for the processing and performance requirements that emerging lignin-based packaging adhesives must meet.

Lignin is particularly attractive because it combines renewable origin with an aromatic, polyphenolic structure that differs substantially from the predominantly aliphatic structures of many other bio-based polymers. Its phenolic and aliphatic hydroxyl groups, aromatic rigidity, and relatively hydrophobic character provide opportunities to enhance cohesive strength, water resistance, thermal stability, and interaction with other adhesive components. In addition, technical lignin is generated in large quantities as a side stream of pulp, paper, and biorefinery processes, creating an opportunity for its valorisation in higher-value adhesive applications. However, lignin is structurally heterogeneous, and its reactivity, solubility, colour, purity, and processing behaviour depend strongly on the biomass source and isolation process [2,6].

Much of the published research on lignin-based adhesives has focused on plywood, particleboard, fibreboard, and other wood products, where lignin is commonly investigated as a partial substitute for phenol in thermosetting resins or as a reactive component in formaldehyde-free systems [7,8,9]. These studies provide important information on lignin modification, crosslinking, curing behaviour, water resistance, and bonding mechanisms. Nevertheless, their results cannot be transferred directly to packaging, where adhesives may be applied at substantially lower coat weights, processed at higher production speeds, and required to bond flexible, coated, or non-porous substrates. Direct evidence concerning lignin-based adhesives for paper, paperboard, labels, films, laminates, glass, and other packaging materials remains comparatively limited.

This review critically examines the potential of lignin-based adhesives for packaging applications. It outlines key packaging-adhesive requirements, briefly positions other bio-based systems as comparative references, and focuses on technical lignin types, modification strategies, and structure–property relationships. Because direct studies on lignin-based adhesives for packaging applications remain limited, relevant findings from wood-bonding research are included where they provide transferable insights, while their applicability to packaging systems is critically assessed. The review further addresses packaging-related applications, processing and safety challenges, industrial scale-up, environmental performance, and recyclability, with the aim of identifying current opportunities and remaining research gaps.

During the preparation of this manuscript, the authors used AI-supported and web-based tools to assist with literature identification, screening, language editing, and translation. Consensus (Consensus NLP, Inc., Boston, MA, USA), a web-based AI academic search platform, was used between January and April 2026, and Google Scholar Labs (Google LLC, Mountain View, CA, USA), a web-based literature-discovery tool, was used between December 2025 and May 2026 to identify and screen relevant scientific articles and abstracts across the research fields addressed in this review. In addition, InstaText (InstaText d.o.o., Slovenia), a web-based AI writing assistant, was used in May 2026, and DeepL (DeepL SE, Cologne, Germany), a web-based AI translation and writing assistant, was used between March and May 2026 to support language editing, translation of English terms and expressions, and overall clarity of the manuscript. All AI-assisted outputs were carefully reviewed, verified, and edited by the authors, who take full responsibility for the content, interpretation, and conclusions presented in this publication.

## 2. Packaging Adhesives: Fundamentals and Requirements

### 2.1. Fundamentals of Adhesion

Adhesion is a fundamental aspect of material behavior that governs interactions between different substances, both in natural systems and in engineered materials. In applied contexts, this phenomenon underpins adhesive bonding, a key area of materials science concerned with joining substrates and forming composite structures. Given the diversity of materials and bonding conditions, adhesion can be described through a range of mechanisms that operate across different interfaces [10].

These mechanisms are generally categorized into two main groups: those based on mechanical interactions, such as interlocking or entanglement, and those driven by physicochemical interactions, including various forms of intermolecular and electrostatic forces. Several theories have been proposed to explain adhesive behavior, including mechanical interlocking, electrostatic interactions, adsorption and wetting, diffusion, covalent bonding, acid–base interactions, and the presence of weak boundary layers [10,11,12].

### 2.2. Requirements for Packaging Adhesives

Adhesive bonding involves the permanent joining of two substrates through the application of an adhesive material. While this process is relatively well understood for dense, non-porous materials, it becomes more complex when applied to porous, fibre-based substrates such as paper and paperboard. In such systems, bond strength is influenced by a combination of substrate characteristics and adhesive properties, including surface structure, absorbency, and interaction at the interface [1].

Paper and paperboard represent some of the most important packaging substrates due to their versatility, recyclability, and broad applicability across the packaging sector. However, packaging applications also rely on a diverse range of other materials, selected according to product requirements, barrier performance, processability, and shelf-life demands. These include metals such as tinplate, tin-free steel, aluminium, and aluminium foils, as well as metallized films including aluminium-coated polypropylene and poly(ethylene terephthalate) (PET). Commodity polymers, including polyethylene, polypropylene, ethylene–acrylate ionomers, polystyrene, poly(vinyl chloride) (PVC), PET, and polyamides, are also extensively used. Glass also remains an important packaging material, particularly in the food and pharmaceutical industries, because it supports product safety, preservation, sensory quality, chemical resistance, recyclability, and diverse design possibilities [13,14].

Both natural and synthetic adhesives are widely used in packaging applications, with their selection depending on performance requirements and processing conditions. Adhesives are typically applied in three main forms: aqueous systems, primarily used in paper and board; carrier-free systems such as hot-melts and reactive liquids, commonly applied in high-speed processes and lamination; and organic solvent-based systems, mainly for flexible laminates and difficult-to-bond surfaces. Natural adhesives remain important due to their low cost and ease of handling, particularly in paper and board applications, although their performance is more limited. In contrast, synthetic adhesives, especially poly(vinyl acetate) (PVAc)-based systems, offer greater versatility and can bond a wider range of substrates, including polymers and coated surfaces. Increasing production speeds and efficiency demands have driven the adoption of hot-melt adhesives, which provide rapid bonding and compatibility with diverse materials [1].

Adhesive selection is also influenced by regulatory and safety requirements, particularly when packaging materials are intended for food contact. The EU framework for food contact materials, now governed by Regulation (EC) No. 1935/2004, sets general safety requirements for all packaging materials, including adhesives. It mandates that such materials must not endanger human health, alter food composition, or affect its taste and smell [15].

## 3. An Overview of Bio-Based Adhesives

Although the primary focus of this review is on lignin-based adhesives, selected classes of other bio-based adhesives are discussed to provide an application-oriented reference framework. Direct investigations of lignin-based adhesives in packaging remain comparatively limited, whereas starch-, protein-, tannin-, and vegetable-oil-based formulations have been more extensively investigated for paper and paperboard bonding, corrugated-board production, labelling, coatings, and flexible packaging [1]. These established systems therefore provide useful information regarding the processing and performance requirements that emerging lignin-based adhesives must meet. Their inclusion is not intended to provide a comprehensive review of all bio-based adhesive families, but to highlight relevant packaging applications and formulation approaches that may inform the development of lignin-containing systems.

### 3.1. Polysaccharide-Based Adhesives

Polysaccharides are polymeric carbohydrates composed of monosaccharide units linked by glycosidic bonds. Their molecular structures are rich in hydroxyl groups, which enable extensive hydrogen bonding, chemical modification, and interactions with polar substrates such as paper and paperboard. Polysaccharides are obtained from a wide range of renewable sources, including plants, algae, marine organisms, and microorganisms. Examples include starch, cellulose, hemicellulose, alginate, chitin, chitosan, dextran, and bacterial exopolysaccharides. Owing to their natural abundance, relatively low cost, biodegradability, biocompatibility, and generally low toxicity, polysaccharides have been widely used in the textile, paper, food, and pharmaceutical industries [16]. Some polysaccharides, particularly chitosan, may also exhibit antimicrobial activity, further expanding their functional potential in packaging applications [17].

#### 3.1.1. Starch

Starch is one of the most important natural adhesives used in paper packaging. It is a plant-derived polysaccharide obtained from sources such as corn, wheat, potato, rice, tapioca, and sago [1]. Its low cost, renewability, and biodegradability make starch a promising alternative to petroleum-based plastics and adhesives, particularly for paper and wood applications. However, native starch is highly hydrophilic because its abundant hydroxyl groups readily interact with water, resulting in poor moisture resistance [18]. Although starch adhesives are widely used in the production of corrugated board and other paper-packaging applications, their poor moisture resistance and limited bonding performance require chemical modification [1]. Various chemical modification strategies including esterification, etherification [19], crosslinking [20], oxidation [21] and grafting [22], have been developed to overcome these limitations. As a result, modified starch systems demonstrate significantly improved performance compared to native starch and are more suitable for industrial adhesive applications.

Starch adhesives are extensively used in the production of corrugated board and other paper products. Modified starch derivatives such as dextrins are commonly used because they are water-soluble and can be formulated with different viscosities for specific applications. These adhesives can be further modified with additives like borax or polyvinyl alcohol to improve tack, viscosity development, and bonding strength [1].

Starch is also highly relevant as a matrix for lignin-containing adhesive systems. The hydroxyl-rich starch structure can interact with lignin through hydrogen bonding and, after chemical modification, through covalent or grafted linkages. In such formulations, lignin may contribute aromatic rigidity, hydrophobicity, thermal stability, and additional cohesive interactions, while starch provides film-forming ability, substrate compatibility, and processability in aqueous systems. A lignin-grafted starch adhesive developed for paper bonding demonstrated improved adhesive stability and bonding strength while retaining the possibility of removal with hot water, supporting paper recovery and recycling [23].

#### 3.1.2. Cellulose

Cellulose, the most abundant natural biopolymer, is another important renewable material used in the development of bio-based adhesives and coatings [24,25,26]. Its hydroxyl-rich structure enables hydrogen bonding, chemical modification, and strong interactions with paper and paperboard substrates. In adhesive formulations, cellulose is commonly used in the form of cellulose derivatives, microcrystalline cellulose, cellulose nanofibrils, or cellulose nanocrystals, where it can improve film formation, mechanical reinforcement, viscosity control, and interfacial bonding [26,27].

Cellulose is also relevant as a reinforcing and reactive component in lignin-containing adhesive systems. Direct lignin–cellulose combinations have also been investigated in fully bio-based adhesive systems; for example quinonoid and epoxidized lignin were crosslinked with aminated cellulose through Schiff-base and epoxy–amine reactions, forming a lignin–cellulose–lignin network. The resulting adhesive achieved dry and wet bonding strengths of approximately 3.20 and 1.73 MPa, respectively [28]. In another approach, lignin was used as a natural binder between native cellulosic fibres during hot pressing. The improvements in mechanical strength and water resistance were attributed to fibre densification and enhanced lignin–cellulose interactions, with the best performance observed at an intermediate lignin content of approximately 6 wt% [29]. However, these studies were mainly performed on wood-based materials and often required high temperature, pressure, or chemical modification. Their direct transfer to packaging therefore remains limited and requires further investigation under paper- and paperboard-relevant processing conditions.

### 3.2. Protein-Based Adhesives

Common plant proteins used for adhesive formulations include soy protein [30], wheat gluten [31], cottonseed protein [32], canola (rapeseed) protein [33], and zein derived from corn [34]. These materials are attractive because of their renewability, biodegradability, and generally good dry adhesion; however, their wider application is often limited by poor water resistance, limited wet stability, and susceptibility to microbial degradation [3,8]. Although this section focuses mainly on plant proteins, casein adhesives remain relevant for glass-bottle and jar labelling because they provide strong initial tack and can retain performance under cold or wet conditions, while allowing label removal in washing solutions [35].

Among plant proteins, soy protein is the most widely used due to its low cost, high availability, and biodegradable nature. Lignin is particularly relevant as a co-component in protein-based adhesives because its aromatic and phenolic structure can contribute additional intermolecular interactions, rigidity, thermal stability, hydrophobicity, and resistance to biological degradation. For example, the incorporation of alkali lignin into a soybean protein adhesive increased wet shear strength to 1.29 MPa, approximately 92.5% higher than that of the unmodified adhesive, while also improving coating behaviour, toughness, and resistance to mould growth [36]. Although this result was obtained for wood bonding, it demonstrates how lignin may help address the moisture sensitivity and limited durability of protein-based adhesives.

### 3.3. Vegetable Oil-Based Adhesives

Vegetable oil-based adhesives have attracted growing interest as sustainable alternatives to conventional petroleum-derived polyurethane systems, particularly for flexible packaging applications [37]. Recent work has demonstrated the use of epoxidized soybean oil [38], rosin [39], and castor oil-derived [40] polyols in the preparation of solvent-free polyurethane adhesives with improved hydrothermal stability and cooking resistance. In these systems, vegetable-oil segments contribute flexibility and hydrophobicity, while rigid bio-based components such as rosin can enhance cohesive strength and thermal stability. However, despite their relevance to flexible packaging [41], many renewable polyurethane adhesive systems have still been evaluated mainly in wood-bonding applications, and direct packaging studies remain comparatively limited [32,40,42].

Lignin offers a complementary strategy for reinforcing vegetable-oil-derived adhesive networks because its aromatic structure and polar functional groups can contribute rigidity, hydrogen bonding, thermal tolerance, and interfacial cohesion. A recent study incorporated homogenized lignin into a plant-oil-based poly(ester amide) through in situ melt polycondensation, producing recyclable vitrimer-like adhesive composites. At a low lignin content of 3 wt%, tensile strength and toughness increased, whereas higher lignin loadings promoted self-aggregation, reduced network homogeneity, and impaired mechanical performance. The resulting adhesive reached a lap-shear strength of 23.8 MPa on stainless steel and maintained adhesion over a broad temperature range and after exposure to acidic, alkaline, saline, and aqueous environments [43]. These findings demonstrate the complementary roles of flexible plant oil-derived segments and rigid lignin domains, but the high processing temperature of 230 °C and the tendency of lignin to aggregate at elevated concentrations remain important barriers to application in conventional high-speed packaging processes.

### 3.4. Polyphenolic Systems

Tannins are among the most promising renewable raw materials for bio-based adhesive production because of their high polyphenol content, natural abundance, and strong reactivity. They are mainly classified into condensed tannins and hydrolysable tannins, with condensed tannins generally showing greater suitability for adhesive applications due to their higher reactivity and better resistance to hydrolysis [44].

Tannin-based adhesives have been widely investigated for wood composites, plywood, particleboard, and laminates, where they can partially or completely replace petroleum-derived phenol in phenol-formaldehyde resins [45]. Their high content of phenolic hydroxyl groups enables strong intermolecular interactions and chemical crosslinking, leading to good bonding strength and improved thermal stability [46]. In addition, tannins can be combined with other bio-based components such as lignin [47], starch [48] and proteins [49] to further improve adhesive performance.

A recent strategy further demonstrated that tannins could serve not only as co-components but also as structural models for improving lignin reactivity. Kraft lignin was depolymerized and subsequently functionalized with phenolic compounds to produce tannin-like lignin with a higher density of accessible phenolic hydroxyl groups. Resorcinol modification increased the phenolic hydroxyl content by 56.6%, bringing the modified lignin closer to tannic acid in functional-group density [50]. When incorporated into a fully bio-based system containing epoxidized soybean oil, malic acid, and tannic acid, the resulting adhesive achieved a lap-shear strength of 3.3 MPa on aluminium and retained 87% of its dry strength after 24 h of water immersion. The improved performance was attributed to the combined effects of covalent crosslinking, hydrogen bonding, and hydrophobic polyphenolic domains. However, the required curing conditions of 180 °C for 2–4 h may limit its applicability in high-speed packaging processes.

Beyond wood-bonding applications, tannin-based formulations have also been investigated directly for paper packaging. In one study, tannic acid–chitosan and shellac–chitosan adhesive formulations were evaluated on commercial packaging papers and papers produced from invasive plant fibres. The addition of tannic acid improved viscosity and adhesive strength, with the tannic acid–chitosan formulation achieving a tensile strength up to 30% higher than that obtained with a commercial poly(vinyl acetate) (PVAc) adhesive. The bio-based formulations also showed promising peel strength and favourable thermal stability, although their performance varied according to the adhesive composition and paper substrate. These findings provide direct evidence of the potential of tannin-based adhesives for paper-packaging applications, although further evaluation under wet conditions and recycling-relevant conditions is required [51].

## 4. Lignin: Structure, Sources and Reactivity

Among renewable aromatic biopolymers, lignin has emerged as one of the most promising candidates for the development of sustainable adhesive systems. Its aromatic structure, natural abundance, and availability from industrial side streams make lignin particularly attractive as a renewable substitute for petroleum-derived phenolic materials in adhesive formulations.

**Figure 1:**
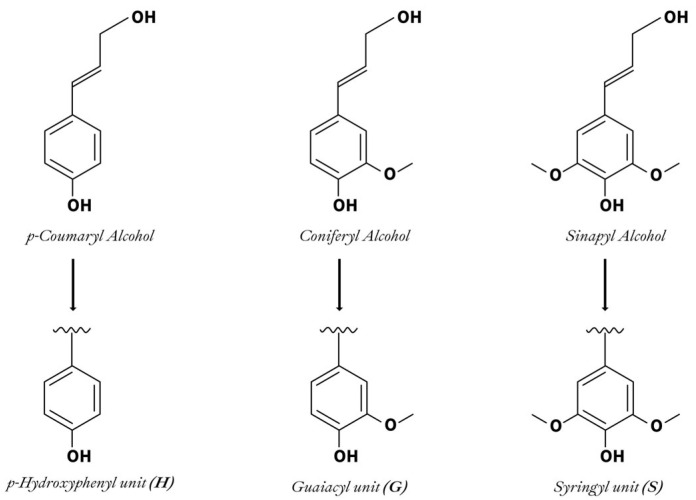
Main monolignol precursors of lignin and their corresponding H, G, and S units.

Lignin is a major structural component of plant secondary cell walls, accounting for approximately 15–36% of plant biomass and representing the second most abundant renewable polymer after cellulose [2]. Unlike cellulose, lignin is not composed of repeating identical monomers but is a highly heterogeneous three-dimensional polymer formed by the oxidative polymerization of three primary monolignols: p-coumaryl, coniferyl, and sinapyl alcohol. These precursors give rise to the p-hydroxyphenyl (H), guaiacyl (G), and syringyl (S) units that constitute the lignin polymer (Figure 1) [52].

The diversity of interunit linkages and reactive functional groups provides numerous possibilities for chemical modification, enabling the design of lignin-based adhesives with tailored physicochemical and bonding properties. The relative abundance of H, G, and S units varies among plant species, influencing both the distribution of interunit linkages and the physicochemical properties of the resulting lignin polymer [8,53].

**Figure 2:**
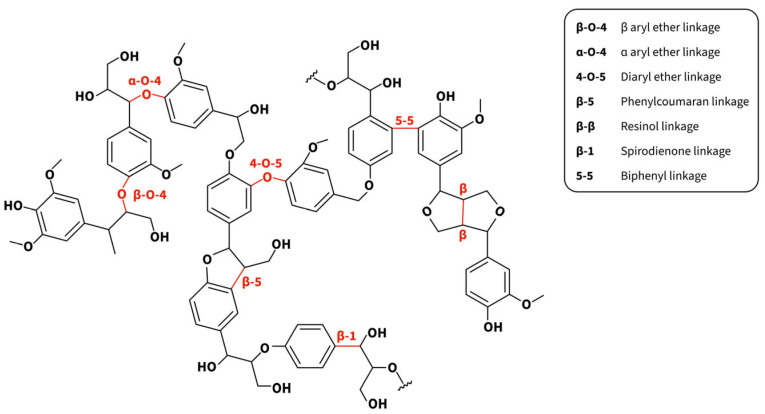
Representative lignin structure showing common interunit linkages.

During lignification, the H, G, and S units are interconnected through radical coupling reactions to form a highly heterogeneous three-dimensional polymer. The lignin macromolecule contains a variety of carbon–carbon and ether linkages, among which the β-O-4 aryl ether bond is usually the most abundant, accounting for approximately 50–60% of interunit linkages depending on lignin source. Other characteristic linkages include β-5, β-β, β-1, 5-5, α-O-4, and 4-O-5 bonds (Figure 2). Together with phenolic and aliphatic hydroxyl groups and other functional groups, these structural features largely determine lignin reactivity and provide numerous opportunities for chemical modification [8,53].

Depending on its origin, lignin is generally classified as native or technical. Native lignin, in its unmodified state, is mainly studied to understand its inherent structure, whereas technical lignin is derived from lignocellulosic biomass or recovered from industrial by-products. Its isolation involves chemically intensive processes, such as acid treatments and pulping, which significantly alter its structure. Depolymerization during processing is often accompanied by condensation, forming additional C–C bonds that increase structural complexity and hinder further utilization. Technical lignin is produced in large quantities, approximately 50–60 million tons annually from the pulp and paper industry and is therefore the primary form used in practical applications [54].

However, the industrial availability of technical lignin does not imply that it represents a uniform or readily interchangeable adhesive raw material. Different biomass sources and isolation processes produce lignins with distinct molecular-weight distributions, degrees of condensation, functional-group contents, sulfur and ash levels, solubility, purity, and colour [8,55]. These structural and compositional differences govern lignin reactivity and directly influence adhesive viscosity, compatibility with other formulation components, curing behaviour, water resistance, and bonding performance [8,56]. The following section therefore compares the principal technical lignin types from a structure–property–performance perspective.

### 4.1. Technical Lignin Types

**Figure 3:**
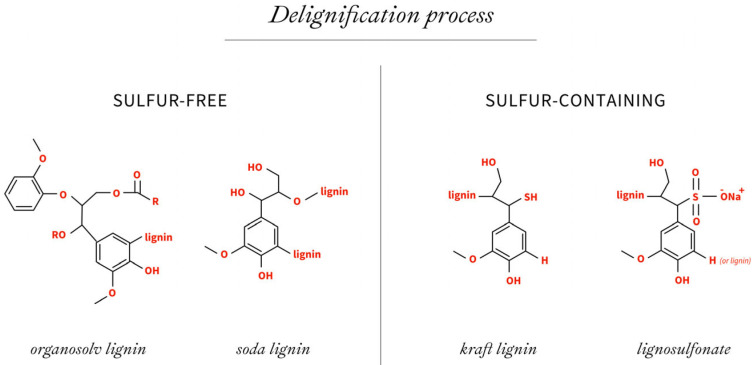
Overview of the technical lignin types (sulfur-free and sulfur-containing process).

Technical lignins are primarily distinguished by the industrial process used for their isolation. Each extraction route modifies the native lignin structure to a different extent, resulting in lignins with distinct molecular characteristics and adhesive performance. Accordingly, industrial delignification processes are generally divided into sulfur-containing and sulfur-free methods (Figure 3). Sulfur-containing processes include kraft and sulfite pulping, whereas sulfur-free processes comprise organosolv and soda pulping. In addition to these conventional technologies, alternative delignification methods such as acid hydrolysis and steam-based treatments have also been investigated [57].

#### 4.1.1. Kraft Lignin

Kraft lignin is one of the most abundant and industrially accessible technical lignins and is recovered from black liquor generated during kraft pulping. Cleavage of aryl ether linkages during pulping increases the content of phenolic hydroxyl groups, which can provide reactive sites for adhesive synthesis. However, concurrent condensation reactions generate additional carbon–carbon linkages and produce a highly altered, heterogeneous, and relatively condensed structure. Kraft lignin also typically contains residual sulfur and exhibits broad molecular-weight distribution, dark colour, and limited solubility in neutral aqueous media. These characteristics provide aromatic rigidity and potential thermal stability, but they may simultaneously restrict functional-group accessibility, compatibility, and curing efficiency [58].

Kraft lignin has been investigated as a partial phenol substitute, reactive adhesive component, and reinforcing binder in a range of resin and composite systems [59]. Its adhesive performance, however, depends not only on the overall hydroxyl content but also on molecular weight, degree of condensation, dispersion, and the accessibility of reactive sites. Consequently, chemical modification or formulation optimization is often required, although the effectiveness of such treatments remains strongly formulation dependent [8]. Kraft lignin therefore offers major advantages in terms of availability and established supply, but its heterogeneity, variable reactivity, and need for formulation-specific optimization remain important barriers to consistent industrial adhesive performance [8].

For packaging applications, the established availability of kraft lignin is advantageous, but its dark color, limited aqueous solubility, variable reactivity, and frequent need for high-temperature curing or chemical modification may restrict its use in fast-setting, waterborne, food-contact, and visually sensitive adhesive systems.

#### 4.1.2. Lignosulfonates

Lignosulfonates are sulfur-containing technical lignins obtained during sulfite pulping, in which sulfur dioxide is used together with calcium, sodium, magnesium, or ammonium bases. The introduction of sulfonate groups gives lignosulfonates an anionic character and high water solubility, which distinguishes them from kraft lignin and facilitates their incorporation into aqueous adhesive systems. Compared with kraft lignin, lignosulfonates generally exhibit a higher average molecular weight and broader molecular-weight distribution, while also containing higher levels of sulfur, ash, residual carbohydrates, inorganic salts, and other process-derived impurities [55].

These characteristics provide both advantages and disadvantages. Their water solubility and surface-active behaviour support dispersion, mixing, and formulation in waterborne systems. However, their hydrophilic nature may increase moisture sensitivity, while differences in counterion, sulfonation degree, molecular weight, purity, and residual carbohydrate content can cause substantial variability in curing and bonding behaviour. For lignosulfonates, performance variability is strongly related to the degree of sulfonation, counterion type, molecular weight, ash content, and residual carbohydrates [8,55].

Reported adhesive performance confirms that lignosulfonates cannot be evaluated solely based on their general classification. In phenol–formaldehyde systems, hydroxymethylated sodium lignosulfonate showed better compatibility than kraft and organosolv lignins despite its higher impurity content, suggesting that the accessibility of reactive hydroxyl groups may be more decisive than purity alone. Conversely, direct incorporation of unmodified sodium or magnesium lignosulfonates into urea–formaldehyde adhesives reduced bonding strength as the substitution level increased, although free-formaldehyde content also decreased [8]. These findings illustrate an important trade-off: lignosulfonates can contribute to lower fossil-derived content and reduced formaldehyde emissions, but mechanical performance may decline if their reactivity and compatibility are not sufficiently controlled.

Chemical activation can partly overcome these limitations. For example, epoxidized sodium lignosulfonate crosslinked with chitosan formed a denser network and improved wet bonding strength compared with the unmodified lignosulfonate–chitosan system. However, the improvement was achieved through additional reagents, a crosslinking component, and hot pressing, while higher lignosulfonate contents increased viscosity and reduced processability [60]. Therefore, modification improves the practical potential of lignosulfonates but also introduces processing and sustainability trade-offs that must be considered for packaging adhesives.

Lignosulfonates offer greater aqueous processability than kraft, soda, and organosolv lignins, making them attractive for waterborne and paper-related adhesive systems. Nevertheless, their hydrophilicity, high sulfur and ash contents, broad molecular variability, and dependence on chemical activation may limit water resistance, reproducibility, and industrial process efficiency. For packaging applications, their water solubility and low-cost availability are advantageous, but other parameters require further evaluation.

#### 4.1.3. Organosolv Lignin

Organosolv lignin is a sulfur-free technical lignin obtained by fractionating lignocellulosic biomass with organic solvents, commonly alcohols, ketones, or organic acids, often in combination with water and a catalyst. Compared with kraft lignin and lignosulfonates, organosolv lignin generally exhibits higher purity, lower ash and carbohydrate contents, lower molecular weight, and greater structural homogeneity [55,61]. Its sulfur-free composition may also reduce odour-related concerns and improve suitability for high-value polymer and adhesive applications [55].

For organosolv lignin, the main source of variability is the extraction severity, including solvent type, catalyst, temperature, and residence time, which determine the preservation or cleavage of β-O-4 linkages and the degree of condensation [61]. Mild extraction conditions may preserve ether linkages and produce relatively reactive lignin, whereas severe acidic or thermal conditions can promote cleavage and recondensation. Consequently, high purity alone does not guarantee high adhesive performance, because compatibility and functional-group accessibility remain formulation-specific [8,61].

Organosolv lignin has also been investigated in pressure-sensitive adhesive formulations, where its hydroxyl groups can interact with ether and carboxylate functionalities of polymer matrices through hydrogen bonding. At moderate lignin contents, these interactions can improve thermal stability and enable adjustment of tack, peel adhesion, and shear resistance, demonstrating the potential of organosolv lignin for higher-value adhesive applications such as labels, coatings, and pressure-sensitive packaging systems [62,63]. However, the effect of lignin content is strongly non-linear. While moderate incorporation can enhance cohesive interactions and creep resistance, excessive lignin loading may restrict polymer-chain mobility, reduce surface wetting, and impair tack because of the rigid and glassy nature of lignin domains [62].

Despite these promising properties, such systems should be regarded as model or base formulations rather than ready-to-use packaging adhesives, because moisture resistance, ageing, removability, migration behaviour, and adhesion to common packaging substrates remain insufficiently evaluated. More broadly, industrial use of organosolv lignin is still limited by solvent recovery requirements, solvent cost and flammability, equipment corrosion, energy demand, and difficulties in maintaining consistent lignin properties during scale-up [55,61].

Organosolv lignin offers important advantages in purity, low sulfur content, structural tunability, and compatibility with selected polymer systems. These properties make it particularly interesting for pressure-sensitive adhesives, coatings, labels, and other higher-value packaging applications. However, its higher production cost and lower commercial availability compared with kraft lignin, together with strong dependence on extraction conditions and formulation composition, remain major barriers to large-scale implementation [55,61].

#### 4.1.4. Soda Lignin

Soda lignin is a sulfur-free technical lignin obtained by alkaline pulping with sodium hydroxide, most commonly from non-wood biomass such as bagasse, wheat straw, grasses, corn stalks, and other agricultural residues. Its lack of sulfur is advantageous for applications in which odour, corrosion, or sulfur-containing residues are undesirable. Soda lignin is generally soluble under alkaline conditions and can contain relatively high levels of phenolic hydroxyl groups, providing potential reactive sites for condensation with aldehydes and incorporation into phenolic adhesive systems [55].

Its molecular weight, hydroxyl-group content, degree of condensation, purity, carbohydrate content, and reactivity depend strongly on the biomass source and pulping severity. Recovery from non-wood black liquor can also be difficult because carboxylic acids and other dissolved components may hinder precipitation, filtration, and centrifugation [55]. Consequently, the performance of soda lignin cannot be predicted solely from its sulfur-free composition.

Soda lignin can provide favourable adhesive performance because its sulfur-free structure and relatively high phenolic hydroxyl content may promote faster curing, stronger network formation, improved bond strength, lower water absorption, and reduced formaldehyde emissions in selected phenolic adhesive systems [63,64]. However, these benefits are formulation- and feedstock-dependent (Table 1). Enhanced reactivity may increase viscosity and reduce processability, while comparative studies show that soda lignin is not consistently superior to kraft or organosolv lignin [56]. For packaging applications, its potential therefore depends on achieving controlled viscosity, moderate curing conditions, colour and odour acceptability, migration safety, and reliable performance on different substrates.

### 4.2. Lignin Modification Strategies

**Figure 4:**
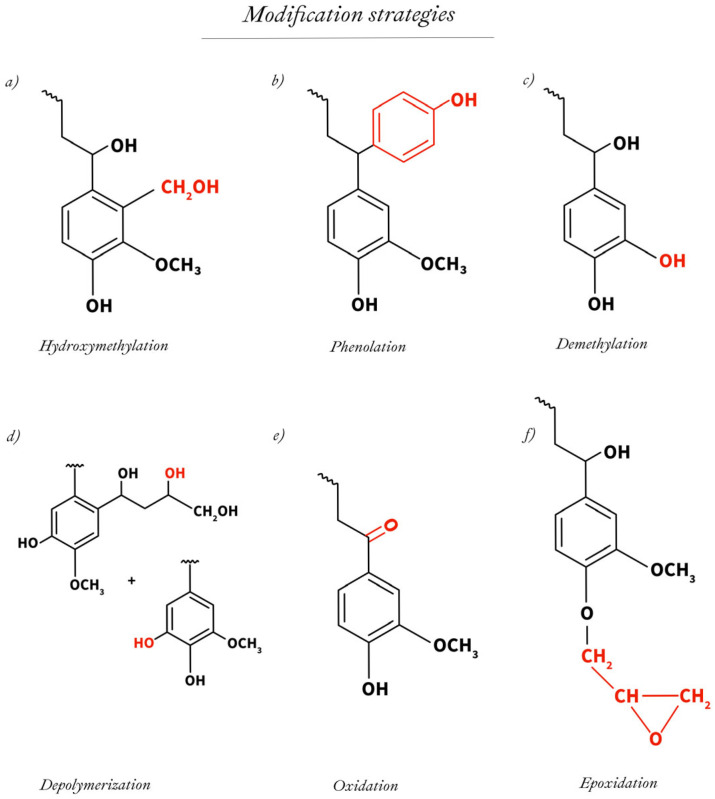
Representative schematic routes for lignin modifications (**a**) hydroxymethilation, (**b**) phenolation, (**c**) demethylation, (**d**) depolymerization, (**e**) oxidation and (**f**) epoxidation.

Although technical lignins offer several advantages as renewable aromatic feedstocks, their direct use in adhesive formulations is often limited by low and variable reactivity, structural heterogeneity, restricted solubility, and limited compatibility with other adhesive components. In many cases, reactive phenolic sites are sterically hindered or reduced by condensation reactions during lignin isolation, which can impair curing efficiency, crosslink formation, and final bonding performance. Chemical modifications are commonly used to increase lignin reactivity, improve functional-group accessibility, enhance solubility or compatibility, and promote stronger adhesive networks. The main modification strategies discussed in this review include hydroxymethylation, phenolation, demethylation, depolymerization, oxidation, and epoxidation (Figure 4). These approaches differ in their chemical mechanisms and practical implications: some introduce new reactive groups, others expose existing functional groups or reduce molecular size, while others enable formaldehyde-free crosslinking reactions. However, modification also introduces additional processing steps, reagents, costs, and potential safety or sustainability trade-offs.

#### 4.2.1. Hydroxymethylation

Hydroxymethylation, also referred to as methylolation, is an established strategy for increasing lignin reactivity by introducing hydroxymethyl groups (–CH_2_OH), mainly at reactive positions adjacent to phenolic hydroxyl groups. These groups can participate in condensation reactions during curing and increase the crosslinking potential of lignin in phenol–formaldehyde and related adhesive systems [70].

Its effectiveness depends strongly on lignin type, molecular weight, degree of condensation, and reactive-site accessibility. Although hydroxymethylation can improve compatibility, curing behavior, bonding strength, and thermal performance, excessive modification may cause premature curing or reduced final strength, indicating that an optimum modification level is required [8,71].

From a packaging perspective, the main drawbacks are the additional reaction step, acidic or alkaline processing conditions, and continued dependence on formaldehyde. Therefore, hydroxymethylation remains useful for improving lignin reactivity, but it is less suitable for applications requiring formaldehyde-free chemistry, low-temperature curing, or strict food-contact safety.

#### 4.2.2. Phenolation

Phenolation is a chemical modification strategy in which phenol or a phenolic derivative is introduced into the lignin structure, most commonly under acidic conditions. This increases the content of phenolic hydroxyl groups and the number of free ortho- and para-reactive sites, thereby improving lignin reactivity toward formaldehyde and its suitability as a phenol substitute in phenol–formaldehyde adhesives [70].

The effectiveness of phenolation depends strongly on the initial lignin structure, including molecular weight, degree of condensation, functional-group accessibility, and the availability of reactive side-chain positions. Lignins with greater initial reactivity may undergo more extensive functionalization, whereas highly condensed lignins can remain less responsive [8]. Phenolation can therefore improve resin reactivity, bonding strength, and formaldehyde consumption, especially when combined with fractionation, but it cannot be considered universally effective for all lignin types [72].

Despite its effectiveness, phenolation presents important sustainability and processing limitations. Conventional approaches may require excess phenol, strong acids, elevated temperature, organic solvents, purification, and repeated washing to remove residual reagents. In addition, many phenolated lignin adhesives remain dependent on fossil-derived phenol and formaldehyde [72]. For packaging applications, these limitations are particularly relevant because residual phenol, formaldehyde, solvents, colour, odour, and migration behaviour may restrict their use.

#### 4.2.3. Demethylation

Demethylation converts aromatic methoxy groups in lignin into phenolic hydroxyl groups, thereby increasing the number of reactive sites and improving lignin reactivity toward formaldehyde and other crosslinking reactions [8,70]. This can enable higher lignin substitution levels in phenolic adhesive systems and may reduce formaldehyde or VOC emissions when performed under mild conditions [73].

However, the effectiveness of demethylation is formulation-dependent. Higher amounts of demethylated lignin can increase viscosity, reduce spreading, and may still result in lower reactivity than phenol. In some systems, demethylation alone improves dry bonding strength but does not provide sufficient wet resistance, requiring additional oxidation or crosslinking steps [73,74].

Demethylation is a useful strategy for increasing lignin reactivity, but its practical value depends on controlling viscosity, avoiding severe or corrosive reagents, simplifying processing, and achieving sufficient wet strength without relying on formaldehyde or additional modification steps.

#### 4.2.4. Depolymerization

Depolymerization reduces lignin molecular size by cleaving ether and carbon–carbon linkages. This can expose additional phenolic hydroxyl groups, reduce steric hindrance, and improve solubility, flowability, dispersion, and compatibility with adhesive components [8,70]. As a result, depolymerized lignin may show improved reactivity, interfacial bonding, and wet bonding performance.

However, the effect of depolymerization depends strongly on treatment severity and adhesive formulation. Excessive degradation can produce highly heterogeneous fragments, reduce cohesive strength, or promote repolymerization and condensation. Some reported improvements also depend on additional crosslinkers, oxidation steps, ultrasound treatment, enzymatic or fungal pretreatment, or hot pressing, making it difficult to attribute performance gains to depolymerization alone [75,76].

Taken together, depolymerization is useful for improving lignin accessibility and compatibility, but its practical value depends on controlling molecular-size reduction, limiting energy and reagent consumption, preventing repolymerization, and ensuring that the resulting lignin fragments provide sufficient cohesion without excessive synthetic crosslinking or high-temperature processing.

#### 4.2.5. Oxidation

Oxidation increases lignin reactivity by converting hydroxyl- and methoxy-containing structures into carbonyl, aldehyde, or quinone groups. These electrophilic groups can react with phenolic, hydroxyl, or amino functionalities and promote the formation of crosslinked adhesive networks. Depending on the oxidant and reaction conditions, oxidation may also cleave ether linkages and reduce lignin molecular size [8,55].

The effectiveness of oxidation depends strongly on controlling the oxidation degree. Moderate oxidation can generate reactive aldehyde or quinone groups and improve crosslinking, whereas excessive oxidation may convert aldehydes into less reactive carboxyl groups, promote side reactions, or reduce adhesive performance [74]. Quinonization is particularly relevant for formaldehyde-free systems because quinone groups can react rapidly with nucleophilic components, especially amino groups [77].

However, oxidation-based systems often depend on oxidants, additional crosslinkers, or carefully balanced formulation conditions. Their packaging relevance therefore depends on controlling by-products, residual oxidants, colour, storage stability, migration safety, low-temperature curing, and performance on thin paper, board, or polymeric substrates.

#### 4.2.6. Epoxidation

Epoxidation introduces reactive epoxy groups into lignin, most commonly through reactions of phenolic or aliphatic hydroxyl groups with epichlorohydrin or multifunctional glycidyl ethers under alkaline conditions. These epoxy groups can undergo ring-opening reactions with amino, hydroxyl, or carboxyl groups, forming crosslinked networks that improve compatibility, cohesive strength, curing efficiency, and water resistance [8].

Epoxidized lignins have been used in formaldehyde-free systems, for example by crosslinking epoxidized lignosulfonates with chitosan or combining epoxidized lignin with cellulose-based components [28,60]. However, the final adhesive performance depends strongly on lignin structure, hydroxyl-group accessibility, epoxidation degree, curing agent, and formulation ratio. Excess epoxidized lignin may reduce performance by increasing hydrophilicity, leaving unreacted groups, or impairing network homogeneity.

The main limitations are the frequent use of epichlorohydrin, glycidyl reagents, catalysts, organic solvents, alkaline conditions, and additional curing agents. For packaging applications, epoxidation is promising for improving wet bonding performance, but residual reagents, migration safety, flexibility, recyclability, and lower-temperature curing still require further evaluation.

Altogether, lignin modification strategies improve adhesive performance through different mechanisms, including the introduction of reactive groups, increased phenolic hydroxyl content, reduced molecular size, improved compatibility, and enhanced crosslinking. However, no single strategy is universally superior. Their practical relevance for packaging adhesives depends not only on bonding strength, but also on processing complexity, reagent safety, curing temperature, viscosity, etc. Future development should prioritize modification routes that combine sufficient adhesive performance with simple processing, low toxicity, and compatibility with packaging substrates and end-of-life requirements.

## 5. Lignin-Based Packaging Applications

Much of the published research on lignin-based adhesives has been conducted in the field of wood bonding, particularly for plywood, particleboard, fibreboard, and other engineered wood products. This emphasis reflects the chemical similarity between lignin and phenolic adhesive components, the established use of thermosetting resins in the wood industry, and the ability of porous lignocellulosic substrates to tolerate relatively high adhesive application amounts and hot-pressing conditions (Table 2). Wood-adhesive studies therefore provide valuable information on lignin reactivity, substitution of fossil-derived phenol, crosslinking mechanisms, water resistance, curing behaviour, and the effects of chemical modification. However, their performance cannot be transferred directly to packaging applications because wood bonding commonly involves thicker adhesive layers, porous rigid substrates, elevated pressing temperatures, and longer curing times. Packaging systems may instead require low coat weights, rapid setting, flexibility, controlled tack and viscosity, compatibility with non-porous substrates, and suitability for recycling or food contact.

The systems summarized above demonstrate the overview of lignin wood adhesives, but they should not be interpreted as direct evidence of suitability for packaging. Their relevance lies primarily in identifying transferable mechanisms and formulation approaches. The following sections therefore focus specifically on studies in which lignin-containing adhesives, coatings, or bonding systems were evaluated on packaging-relevant substrates or for functions associated with paper, paperboard, labels, films, laminates, glass, or other packaging materials.

Current research focuses on lignin incorporation into paper and paperboard systems [83], food-contact coatings [84], and thermoplastic or hot-melt adhesive formulations [85], with the aim of achieving biodegradable materials that also provide enhanced barrier performance and additional functional properties, such as antioxidant and antimicrobial activity (Table 3). Lignin has generally been reported to exhibit low cytotoxicity at relevant concentrations, although its biological response depends strongly on source and isolation method, supporting its potential use in packaging applications where material safety is essential [86]. Although films and coatings are not necessarily adhesives, these studies provide relevant information on lignin compatibility, dispersion, film formation, interfacial interactions, moisture resistance, and barrier performance.

For example, lignin-based hot-melt adhesive formulations for paperboard achieved bond strength comparable to or higher than a commercial HMA reference, with the best formulation reaching 16.1 N compared with 10.5 N for the reference adhesive [87]. In paper-coating systems, esterified lignin incorporated into PBAT improved wet tensile properties and enhanced water, oil, and oxygen barrier performance, indicating that lignin modification can improve both dispersion in polymer matrices and packaging-relevant barrier properties [84]. Similarly, starch/lignin nanoparticle films showed improved mechanical strength, thermal stability, antioxidant activity, UV-shielding performance, and reduced oxygen permeability, supporting the role of lignin as a functional filler in bio-based packaging matrices [85].

Building on this, lignin-based nanocomposites have been explored as advanced functional materials. For example, lignin and lignin-derived CuO nanoparticles incorporated into polyvinyl alcohol (PVA)–polyethylene glycol (PEG) matrices yield biodegradable films with improved mechanical and thermal properties, as well as antimicrobial, antioxidant, and UV-blocking performance, demonstrating strong potential for active packaging applications [88]. Similarly, lignin nanoparticles (LNPs) have emerged as effective bio-based nanofillers for enhancing biodegradable polymer films. Their incorporation into starch matrices improves mechanical strength, thermal stability, and barrier properties through strong interfacial interactions and uniform dispersion, while also imparting antioxidant and UV-shielding functionality and reducing oxygen permeability [87].

### Lignin-Based Adhesives for Paper and Paperboard

Despite the growing interest in lignin as a sustainable material, its application as an adhesive in packaging systems remains relatively underexplored. Most lignin-based adhesive research has focused on wood-based products, whereas studies specifically targeting paper, paperboard, labels, and other packaging substrates remain limited. The available examples indicate two promising directions: waterborne paper adhesives based on lignin–polysaccharide systems and thermoplastic hot-melt adhesives for paperboard.

Nevertheless, some recent studies demonstrate the emerging potential of lignin in packaging adhesives. For example, lignin has been incorporated into starch-based systems to develop fully bio-based paper adhesives with improved bonding strength and stability, while also enabling recyclability through water-assisted removal [23]. Such systems rely on grafting lignin onto starch backbones to enhance adhesion and maintain biodegradability, highlighting a promising direction for sustainable packaging solutions.

The resulting adhesive provided effective bonding to paper and could be dissolved and removed using hot water, facilitating separation of the adhesive during paper recovery. This behavior is particularly relevant to packaging because adhesives that remain as insoluble residues may form deposits and interfere with paper-recycling equipment. However, removability in hot water should not be regarded as complete proof of recyclability, which must be evaluated under standardized repulping, screening, and adhesive-residue testing conditions.

In addition, lignin and lignin derivatives have been successfully formulated into hot-melt adhesives for paperboard applications, where they can achieve bonding performance comparable to or exceeding that of commercial adhesives, while enabling tunable properties such as reversibility and reduced reliance on external plasticizers. The best-performing formulation, consisting of oxidized cellulose acetate, organosolv lignin, and triethyl citrate, reached a bond strength of 16.1 N, compared with 10.5 N for the commercial hot-melt adhesive used as a reference [85]. Modification of lignin with tall-oil fatty acids also enabled the preparation of a fully bio-based formulation without an external plasticizer, which achieved a bond strength of 8.9 N. The selection and modification of lignin allowed the adhesive properties to be adjusted towards either reversible or irreversible bonded seams.

However, these examples remain relatively scarce, underscoring the need for further research into lignin-based adhesive formulations specifically designed for packaging applications. Its wider industrial use remains limited by low intrinsic reactivity, structural heterogeneity, and variable composition. These factors can lead to inconsistent curing behavior and adhesive performance, with some lignin-based systems still showing lower strength, durability, or water resistance than conventional synthetic adhesives. Additional challenges, including dark color, possible odor, the use of formaldehyde-containing systems in some formulations, and high modification or processing costs, further limit large-scale adoption. Therefore, further advances in lignin modification, cost-effective processing, and formulation optimization are needed to improve performance and support broader industrial application.

## 6. Challenges and Industrial Implementation of Lignin-Based Adhesives in Packaging

### 6.1. Lignin Heterogeneity, Reproducibility, and Processability

Lignin’s structure depends on the botanical source, plant species, growing conditions, and particularly the isolation or pulping process. Consequently, technical lignins differ in molecular-weight distribution, degree of condensation, functional-group content, solubility, and the presence of sulfur, carbohydrates, ash, and other impurities. Considerable variability may also occur among batches classified as the same technical lignin, making the general term “lignin” insufficient to predict adhesive behavior without detailed characterization [8,70].

As discussed, differences among technical lignins directly affect adhesive formulation and processing. Variations in molecular architecture, branching, phenolic hydroxyl content, and reactive-site accessibility influence dispersion, viscosity, curing behavior, and compatibility with the surrounding adhesive matrix. Direct comparisons of different lignins in phenol–formaldehyde systems have shown substantial differences in resin viscosity and curing performance, even at comparable phenol-replacement levels [56]. Similarly, soda- and kraft-lignin-containing adhesives produced different gel times, curing temperatures, panel properties, dimensional stability, and formaldehyde emissions [63]. These findings demonstrate that adhesive performance cannot be predicted from lignin content alone.

Fractionation can partially reduce lignin heterogeneity by producing fractions with narrower molecular-weight distributions and more consistent functional-group contents. However, fractionation introduces additional separation steps, solvent consumption, cost, and potential material losses, and may still require further chemical modification to achieve satisfactory adhesive performance [72].

Industrial reproducibility therefore requires lignin specifications that extend beyond biomass source and pulping category. Relevant quality parameters should include molecular-weight distribution, phenolic hydroxyl content, degree of condensation, solubility, moisture, ash, sulfur, carbohydrate content, and viscosity under defined formulation conditions [8,55]. Standardized characterization procedures and acceptable batch-to-batch ranges are essential for reliable processing, particularly in packaging applications.

### 6.2. Performance and Processing Limitations

Despite considerable improvements in lignin-based adhesive performance, several limitations continue to restrict their transition from laboratory research to industrial production. A central challenge is the relatively low intrinsic reactivity of many technical lignins. Consequently, high lignin-substitution levels may increase viscosity, alter curing kinetics, reduce crosslink density, and impair adhesive spreading or substrate penetration. In phenol–formaldehyde systems, lignin incorporation may also increase the proportion of formaldehyde remaining after resin synthesis and require higher curing temperatures than conventional formulations [8,56].

Chemical modification can overcome some of these limitations, but improved adhesive performance is frequently achieved at the expense of formulation simplicity and environmental compatibility. Hydroxymethylation and phenolation may retain dependence on formaldehyde and fossil-derived phenol, while epoxidation commonly requires epichlorohydrin or multifunctional glycidyl reagents. Oxidation, depolymerization, and grafting often involve harsh reaction conditions, additional purification, and energy-intensive processing. Furthermore, many high-performing formaldehyde-free systems still depend on separately synthesized crosslinkers or several complementary modification steps. A formulation may contain predominantly renewable adhesive components while its overall production route remains chemically complex and dependent on non-renewable reagents [28,77].

Performance trade-offs also remain evident. Increasing lignin content or reactivity can improve bonding strength and water resistance but may simultaneously increase viscosity, brittleness, premature gelation, or sensitivity to formulation ratios. Excessive lignin loading or modification can reduce flow, substrate wetting, and penetration, whereas insufficient crosslinking may result in poor wet strength and limited durability [43]. The dark color of lignin and possible odor may further restrict its use in visually or sensorially sensitive products. In addition, results obtained under optimized laboratory conditions are difficult to compare because studies use different substrates, coat weights, pressing conditions, conditioning procedures, and strength tests. Long-term ageing, cyclic humidity, storage stability, creep, and performance under realistic service conditions are also examined less frequently than initial dry or wet bonding strength [8].

Scalability and economic viability remain constrained by multi-step extraction, modification, purification, solvent recovery, and drying, which increase energy demand, material losses, and production costs. Some proposed systems require hot pressing at elevated temperatures for several minutes, whereas industrial packaging lines generally demand rapid setting, controlled viscosity and continuous application. Laboratory-scale performance must be validated through pilot-scale and demonstration studies, accompanied by techno-economic and life-cycle assessments. Reliable supply chains, recovery of solvents and catalysts, and consistent quality specifications are also required before lignin-derived adhesive components can compete with established commercial systems [8,55,61].

### 6.3. Safety, Regulatory, and End-of-Life Considerations

For food-packaging applications, the renewable origin of lignin does not by itself demonstrate the safety of the resulting adhesive. Food-contact suitability must be evaluated for the complete formulation, including the technical lignin, modification reagents, crosslinkers, plasticizers, preservatives, catalysts, and any reaction by-products. Depending on the lignin type and modification route, potentially relevant substances may include low-molecular-weight lignin fragments, residual phenol or formaldehyde, solvents, epichlorohydrin or glycidyl compounds, oxidants, and other process-derived impurities. Their presence and migration behavior depend on purification efficiency, curing conditions, adhesive coat weight, packaging structure, food type, contact time, and temperature [86].

For food-contact applications, potential migration of residual monomers, crosslinkers, catalysts, solvents, and reaction or degradation products must be assessed to ensure that the finished material does not endanger human health, cause an unacceptable change in food composition, or adversely affect its organoleptic properties, in accordance with Regulation (EC) No 1935/2004 (23). Adhesive residues can form problematic contaminants, commonly referred to as stickies [89], which are difficult to remove during recovered-paper processing and may interfere with pulp and paper production. The repulpability, deinking behaviour, and contaminant formation of lignin-based adhesives should therefore be specifically evaluated. Industrial implementation requires more than maximizing laboratory bonding strength. Promising systems should combine sufficient dry and wet adhesion with simple aqueous preparation, high solids content at manageable viscosity, rapid curing at moderate temperature, safe and commercially available components, and stable performance across different lignin batches and packaging substrates. Future studies should use application-specific benchmarks and standardized testing, report complete formulation and processing data, and integrate performance evaluation with safety, economic, and environmental assessments. Such an approach would enable lignin-based adhesives to be evaluated as complete industrial systems rather than solely as high-performing laboratory formulations.

## 7. Environmental and Sustainability Aspects of Lignin-Based Adhesives

The bio-based origin of an adhesive does not automatically ensure lower environmental impacts than those of a conventional petroleum-derived system. A systematic review comparing bio-based and petrochemical adhesives identified a substantial lack of direct environmental assessments. Although 563 publications were initially screened, only 11 studies evaluated the environmental impacts of the adhesives themselves rather than those of the resulting composite products. Most available studies applied life-cycle assessment (LCA), but differences in system boundaries, functional units, allocation procedures, and impact assessment methods limited direct comparison [90]. Direct environmental assessments of lignin-based adhesives intended specifically for packaging remain particularly scarce; consequently, findings obtained from wood-adhesive systems should primarily be interpreted as indicators of potential environmental benefits and process hotspots rather than as direct evidence for packaging applications.

Lignin offers potential environmental benefits because it is generated in large quantities as a side stream of the pulp, paper, and emerging biorefinery industries and can partially replace fossil-derived adhesive components. In phenolic and urea–formaldehyde systems, lignin substitution may reduce the consumption of petrochemical phenol and other fossil resources and, under suitable conditions, lower greenhouse-gas emissions [8,91,92]. However, these benefits depend on whether lignin is treated as a residue, by-product, or co-product, as well as on the environmental burdens allocated to its separation, purification, and subsequent processing. The use of lignin should therefore not be considered environmentally beneficial solely on the basis of its renewable carbon content.

Published LCA results for lignin-based adhesives are not uniform. Some systems have shown higher environmental impacts than both other bio-based and conventional adhesives, whereas others have achieved more favourable results, including reductions in overall environmental impact of up to approximately 22% for kraft-lignin- or lignosulfonate-containing formulations compared with urea–formaldehyde adhesives [67]. The outcome is strongly influenced by the lignin source and isolation process, substitution level, adhesive composition, allocation method, treatment of biogenic carbon, energy mix, and selected functional unit [67,92]. Comparisons based only on the mass of adhesive may also be insufficient when formulations differ in application amount, curing energy, durability, or required bonding performance. Environmental assessments should therefore reflect the intended adhesive function and service conditions.

Chemical modification represents an additional environmental trade-off. Treatments that improve lignin reactivity, solubility, compatibility, and bonding performance may require considerable quantities of reagents, solvents, water, and energy. Glyoxalation, for example, has been identified as a potential environmental hotspot because of its electricity demand and processing conditions [67]. Similarly, energy-intensive lignin extraction, solvent recovery, purification, drying, and functionalization can offset part of the advantages associated with renewable feedstocks, particularly in some organosolv-based and highly modified systems [93,94,95]. Synthetic crosslinkers and other fossil-derived formulation components must also be included when determining the overall bio-based content and environmental profile of the adhesive.

For packaging applications, LCA and recyclability should be treated as complementary but distinct aspects of environmental performance. A favourable life-cycle profile does not necessarily imply good recyclability, as even a low-impact adhesive may interfere with fibre recovery, separation operations, washing, deinking, or the quality of the resulting recyclate. Under the European packaging framework, recyclability is increasingly addressed as a design requirement for the complete packaging system rather than as a property of the principal substrate alone [96].

At present, the environmental relevance of lignin-based packaging adhesives remains difficult to quantify because the most directly relevant studies rarely include life-cycle or techno-economic assessments. For example, lignin–starch paper adhesives, lignin-based hot-melt adhesives, and lignin-containing coatings demonstrate promising bonding, removability, or barrier performance, but their environmental benefits cannot be confirmed without considering adhesive dosage, curing energy, chemical modification, residual reagents, recyclability, and comparison with commercial reference systems. Consequently, lignin-based adhesives should be evaluated as complete formulations and packaging systems rather than as sustainable materials solely because they contain renewable lignin.

## 8. Future Perspectives

Despite progress, lignin-based adhesives still face challenges related to heterogeneity, variable reactivity, inconsistent performance, and limited industrial processability [8]. Future research should move beyond maximizing bonding strength under laboratory conditions and focus on the combined requirements of adhesive performance, processability, safety, cost, and environmental compatibility.

Lignin-based adhesives and coatings show strong potential for sustainable functional packaging applications, particularly in active and barrier food packaging. However, their broader implementation is still limited by several challenges, including structural variability, limited compatibility with other matrix materials [86], insufficient food safety data [97], and difficulties in scaling up and standardizing production processes.

For packaging applications, more direct studies are required on paper, paperboard, labels, coated substrates, polymer films, aluminum, and glass. Future formulations should be evaluated under packaging-relevant conditions, including low coat weights, rapid application and setting, humid or refrigerated storage, temperature fluctuations, ageing, and contact with water or packaged products. Their performance should also be compared with established commercial packaging adhesives using consistent test methods.

Food-contact safety and end-of-life compatibility require particular attention. Future studies should assess the migration of low-molecular-weight lignin fractions, residual modification reagents, crosslinkers, catalysts, and reaction by-products from the complete cured formulation. Recyclability claims should be supported by testing of the final packaging construction under relevant repulping, screening, adhesive-residue, and fibre-recovery procedures rather than inferred from the renewable origin or hot-water solubility of individual components.

Finally, the transition from laboratory formulations to industrial products will require pilot-scale trials, long-term processing studies, techno-economic assessment, and transparent life-cycle evaluation. These measures will help determine which lignin types and modification strategies can provide practical improvements over established packaging adhesives rather than merely demonstrating technical feasibility at laboratory scale.

## 9. Conclusions

The growing demand for sustainable packaging materials has intensified interest in bio-based adhesives as alternatives to conventional petroleum-derived systems. This review highlights the broad range of renewable adhesive materials currently under investigation, including polysaccharide-, protein-, vegetable oil-, tannin-, and lignin-based systems, with particular emphasis on the emerging role of lignin in advanced adhesive technologies. While starch- and protein-based adhesives offer advantages such as biodegradability, renewability, and low toxicity, their practical application is often limited by poor water resistance, lower mechanical performance, and sensitivity to environmental conditions. In contrast, lignin provides unique benefits due to its aromatic structure, hydrophobic character, and abundance as an industrial by-product, making it a promising platform for the development of high-performance sustainable adhesives.

Recent advances demonstrate that lignin can serve not only as a partial replacement for petrochemical phenolic compounds but also as an active structural and functional component in adhesive systems. Various chemical modification approaches, including oxidation, grafting, epoxidation, and enzymatic activation, have significantly improved lignin reactivity and enabled the development of adhesives with enhanced mechanical strength, thermal stability, and water resistance. In addition, lignin has shown strong potential in packaging-related applications beyond conventional wood adhesives, particularly in coatings, nanocomposites, barrier materials, and emerging paper-based adhesive systems. The incorporation of lignin into packaging materials can also provide additional functionalities, such as antioxidant activity, UV protection, and improved barrier performance, which are highly relevant for active and sustainable packaging technologies.

From an environmental perspective, lignin-based adhesives generally offer reduced dependence on fossil resources and lower greenhouse gas emissions compared with conventional petrochemical systems. However, the sustainability benefits remain strongly dependent on lignin source, extraction route, modification chemistry, and processing conditions. Energy-intensive functionalization methods and the use of certain crosslinkers or solvents may partially offset the environmental advantages of renewable feedstocks. Furthermore, the lack of standardized life-cycle assessment methodologies and limited industrial-scale data currently complicate direct comparisons between adhesive systems.

Despite substantial progress, several important challenges remain before lignin-based adhesives can achieve widespread commercial adoption in packaging applications. These include the intrinsic structural variability of lignin, limited compatibility with some polymer matrices, insufficient food-contact and toxicological data, and difficulties associated with process scalability and standardization. Future research should therefore focus on developing more efficient and environmentally benign lignin modification strategies, increasing lignin utilization in adhesive formulations, improving recyclability and biodegradability, and establishing comprehensive assessments of safety, performance, and life-cycle impacts. Overall, lignin represents a highly promising renewable resource for next-generation sustainable adhesives and functional packaging materials, with significant potential to contribute to the transition toward a more circular and bio-based packaging industry.

## Figures and Tables

**Table 1:** Main lignin types and their relevance for adhesive applications.

Lignin Type	Source	Process	Key Features	Relevance	Ref.
Kraft lignin	Production of pulp	Kraft process	High phenolic OH, condensed, sulfur-containing, lower molecular weight	Most abundant, widely studied for adhesives, good for phenolic resins	[63,65]
Lignosulfonates	Lignocellulose biomass	Sulfite pulping	Water-soluble, high molecular weight	Used in dispersants, limited structural applications	[66,67]
Organosolv lignin	Bamboo, agricultural residue	Organic solvent extraction	Sulfur-free, low molecular weight, more homogeneous	High purity, suitable for high-performance materials	[53,68]
Soda lignin	Lignocellulose biomass	Pulping	Sulfur-free, less condensed than kraft	Often used for non-wood biomass	[57,69]

**Table 2:** Selected lignin-based adhesive systems investigated primarily for wood bonding.

Adhesive Application	Adhesive Type	Lignin Type	Mechanical Properties	Potential Relevance for Packaging	Ref.
Wood adhesive	Soybean protein adhesive	Alkali lignin (AL)	The wet shear strength reached 1.29 MPa, which is 92.5% higher than that of the original adhesive	Suitable viscosity, superior coating properties, and toughness, anti-bacterial and antimould properties	[36]
Hyperbranched adhesive	Lignin-graft acrylic copolymer adhesive	Organosolv lignin (OL)	Maximal adhesive force and strength up to 34.0 N and 367.8 N·s, respectively (better than the commercial 3 M double-sided adhesive)	High adhesive strength, water-resistance and high UV protection (coatings for outdoor decorative materials) and good solubility. Self-healing behavior	[78]
Wood adhesive	All-lignin adhesive	Softwood kraft lignin (SL), alkali lignin, enzymatic lignin and hydrolysate lignin	Under optimized conditions the adhesive achieved dry and wet bonding strengths of 1.79 ± 0.21 MPa and 0.99 ± 0.13 MPa	Significantly enhancing hydrophobicity and thermal stability with mechanical interlocking	[79]
Wood adhesive	Bio-based formaldehyde-free adhesive	Wheat straw lignin	Wet shear strength: RLA = 0.66 ± 0.12 MPa; PLA = 0.79 ± 0.11 MPa; OLA = 1.07 ± 0.22 MPa. The OLA adhesive exceeded the standard requirement (>0.7 MPa). Wet residue rate for OLA: 90.39 ± 2.24%)	Formaldehyde-free; environmentally friendly DES extraction; improved water resistance and thermal stability; good dispersion properties; valorization of agricultural waste	[80]
Wood adhesive	Bio-based lignin–epoxy hybrid nanoparticle adhesive	Softwood Kraft lignin	The optimized adhesive achieved competitive adhesive strengths of approximately 5.4 MPa dry strength and 3.5 MPa wet strength in wood bonding tests.	High solvent and alkaline resistance; stable lignin nanoparticles under harsh conditions; waterborne formulation; strong dry/wet adhesion; improved thermal stability; reduced need for lignin prefractionation or chemical pretreatment; environmentally friendly and bio-based adhesive system.	[81]
Wood adhesive	Tannin–lignin bio adhesive	Ethanol organosolv alfa grass lignin	Adhesives containing 10–30% organosolv lignin improved adhesive performance. The optimized formulation containing 50% glyoxalated alfa lignin and 50%. Aleppo pine tannins achieved an internal bond (IB) strength of 0.45 MPa for particleboards (meeting interior-grade panel standards)	Formaldehyde-free; improved particleboard bonding performance; effective use of agricultural biomass-derived lignin; adhesive properties strongly tunable through lignin extraction conditions; compliance with interior-grade particleboard standards.	[82]

**Table 3:** Examples of lignin roles in packaging-related applications.

Application Type	Role of Lignin	Lignin Type	Key Advantages	Ref.
Coating material for paperboard	Improving mechanical and barrier properties	Kraft lignin	Prominent light-stopping and UV-absorbing ability	[83]
Food-contact coatings	Improving mechanical and barrier properties	Kraft lignin	Excellent wet tensile properties and enhanced water, oil, and oxygen barrier performance	[84]
Bio-based nanofillers	Enhanced mechanical properties, thermal stability, antioxidant activity	Lignin nanoparticles	Outstanding UV shielding performance, oxidation delay	[87]
Fully bio-based paper adhesives	Improved mechanical properties	Sodium lignosulphonate	Improved adhesive stability; higher bonding strength; adhesive can be excluded by hot water without affecting the recovery of paper	[23]
Paperboard packaging	Tackifier and thermoplastic bio-based adhesive component	Kraft lignin, Organosolv lignin, Soda lignin	High bond strength compared to commercial hot melt adhesive (HMA)	[85]

## Data Availability

No new data were created or analyzed in this study. Data sharing is not applicable to this article.

## Abbreviations

The following abbreviations are used in this manuscript:

AL	alkali lignin
CAGR	compound annual growth rate
CMC	carboxymethyl cellulose
CuO	copper oxide
DES	deep eutectic solvent
EC	European Community
EU	European Union
GB/T	Chinese national standard
HMA	hot-melt adhesive
IB	internal bond
LCA	life-cycle assessment
LNPs	lignin nanoparticles
OH	hydroxyl group
OL	organosolv lignin
OLA	Oxidized lignin adhesive
PBAT	poly(butylene adipate-co-terephthalate)
PEG	polyethylene glycol
PET	poly(ethylene terephthalate)
PF	phenol–formaldehyde
PLA	phenolated lignin-based adhesive
PVA	poly(vinyl alcohol)
PVAc	poly(vinyl acetate)
PVC	poly(vinyl chloride)
RLA	recovered lignin-based adhesive
SL	softwood kraft lignin
UV	ultraviolet
VOCs	volatile organic compounds
